# Editorial Note

**Published:** 1947

**Authors:** 


### Editorial Note

**Nursing.** Many changes have been made in the conditions of nursing. For the trained nurse the most important are a forty-eight-hour week; no "domestic duties"; the Rushcliffe scale of payments; and permission to live out—supposing the nurse can find anywhere so to live. For the nurse in training a serious effort has been made to make life in the Nurses' Home as much as possible like that of an undergraduate in a University Hostel. There are difficulties. For one thing, the forty-eight-hour week has meant a very considerable increase in the number of nurses required for every hundred beds: and the impossibility of building delays both the extensions that this makes necessary and additional improvements planned before the war and still waiting to be effected. But, at the Bristol Royal Hospital, for example, the Nurses' Home has been placed in the charge of a Warden and her staff of assistants: so that when off duty the nurse may feel "quite free from the hospital." An entertainments officer has been appointed and a hall secured for dances, meetings, acting, etc. Since the General Nursing Council has decided to recognize for training only the larger hospitals with an adequate tutorial staff, the duty falls on these to provide not only for the nursing in their own wards, but trained staff for the smaller hospitals as well. The need is urgent and it is to be hoped that improved conditions will encourage more girls to undertake this essential service.

